# Diversity and Adaptation of Human Respiratory Syncytial Virus Genotypes Circulating in Two Distinct Communities: Public Hospital and Day Care Center

**DOI:** 10.3390/v4112432

**Published:** 2012-10-24

**Authors:** Luiz Gustavo Araujo Gardinassi, Paulo Vitor Marques Simas, Deriane Elias Gomes, Caroline Measso do Bonfim, Felipe Cavassan Nogueira, Gustavo Rocha Garcia, Claudia Márcia Aparecida Carareto, Paula Rahal, Fátima Pereira de Souza

**Affiliations:** 1 Universidade Estadual Paulista, Instituto de Biociências, Letras e Ciências Exatas de São José do Rio Preto, SP. Departamento de Biologia - Rua Cristóvão Colombo, 2265, Jardim Nazareth – Cep: 15054-000, Brazil; Email: simaspvm@yahoo.com.br (P.V.M.S.), carolbonfim@yahoo.com.br (C.M.B.), cavassan@yahoo.com.br (F.C.N.), carareto@ibilce.unesp.br (C.M.A.C.), rahalp@yahoo.com.br (P.R.); 2 Universidade Estadual Paulista, Instituto de Biociências, Letras e Ciências Exatas de São José do Rio Preto, SP. Departamento de Física - Rua Cristóvão Colombo, 2265, Jardim Nazareth – Cep: 15054-000 Brazil; Email: deribela@yahoo.com.br (D.E.G.); 3 Universidade de São Paulo, Faculdade de Medicina de Ribeirão Preto, SP. Departamento de Bioquímica e Imunologia – Av. dos Bandeirantes, 3900 Monte Alegre – Cep: 14049-900 Brazil; Email: gugard@gmail.com (L.G.A.G.), gugarg10@usp.br (G.R.G.)

**Keywords:** respiratory syncytial virus, attachment protein (G), genetic variability, O-glycosilation, selection pressure.

## Abstract

HRSV is one of the most important pathogens causing acute respiratory tract diseases as bronchiolitis and pneumonia among infants. HRSV was isolated from two distinct communities, a public day care center and a public hospital in São José do Rio Preto – SP, Brazil. We obtained partial sequences from G gene that were used on phylogenetic and selection pressure analysis. HRSV accounted for 29% of respiratory infections in hospitalized children and 7.7% in day care center children. On phylogenetic analysis of 60 HRSV strains, 48 (80%) clustered within or adjacent to the GA1 genotype; GA5, NA1, NA2, BA-IV and SAB1 were also observed. SJRP GA1 strains presented variations among deduced amino acids composition and lost the potential O-glycosilation site at amino acid position 295, nevertheless this resulted in an insertion of two potential O-glycosilation sites at positions 296 and 297. Furthermore, a potential O-glycosilation site insertion, at position 293, was only observed for hospital strains. Using SLAC and MEME methods, only amino acid 274 was identified to be under positive selection. This is the first report on HRSV circulation and genotypes classification derived from a day care center community in Brazil.

## 1. Introduction

Human Respiratory Syncytial Virus (HRSV) is a major viral agent causing serious respiratory tract diseases in the pediatric population worldwide [1]. Of the estimated 2 million children under the age of 5 years who require care for HRSV infections annually, 78% are over the age of 1 year [2], although it has been recognized as a main cause of morbidity in children under 1 year of age [3]. HRSV infection results in several outcomes, ranging from common cold-like symptoms to more severe bronchiolitis and pneumonia in children, immunocompromised and elderly individuals [4,5].

HRSV is classified into the *Pneumovirus* genus of the *Paramyxoviridae* family and is composed of an envelope with a negative-sense single-stranded RNA genome, which encodes for 11 proteins. Based on reactions with monoclonal antibodies against the G and F glycoproteins, beyond molecular differences in several genes [6,7], two major groups, HRSVA and HRSVB, have been described [7,8,9,10]. Several HRSVA genotypes were identified in different geographical regions, which include GA1 to GA7 [9,10], SAA1 (South Africa, A1) [11], NA1 and NA2 [12], and most recently ON1 [13]. Thirteen HRSVB genotypes are currently known and designated as GB1 to GB4 [9], SAB1 to SAB3 (South Africa) [11], and BA1 to BA6 (Buenos Aires) [14]. Therefore, the antigenic variability of HRSV strains has been a relevant subject on discussions of the key features contributing to the ability of the virus to re-infect people and cause large-scale yearly outbreaks [15].

The sequence variability of the attachment (G) protein gene, which shows the largest antigenic and genetic differences between the two HRSV groups [16], is commonly used for genotyping HRSVA and HRSVB viruses [13]. The G protein is a type II glycoprotein of 289 to 299 amino acids in length, consisting of the cytoplasmic tail (amino acids [AAs] 1–38), transmembrane domain (AA 38–66), and the ectodomain (AA 66–298) [17]. The C-terminal ectodomain of G protein is comprised of two variable regions, separated by a highly conserved region between amino acids 164 and 176 assumed to represent a receptor-binding site [17]. The two variable regions of the ectodomain contain high serine and threonine residues, which are potential acceptor sites for O-linked sugars affecting the antigenic structure of the G protein as well as impacting on virus infectivity [18,19].

Few studies have evaluated the epidemiology and HRSV genotypes circulating in São Paulo State, Brazil [20,21,22,23,24,25,26] and furthermore this is the first report on HRSV genotypes isolated from children attending a day care center in Brazil. Accordingly, we aimed to identify HRSV strains features by analyzing the genetic variability in the second hypervariable region of the attachment (G) gene of viruses isolated from clinical samples collected in a public day care center, and a public hospital in São José do Rio Preto-SP (SJRP), Brazil. Phylogenetic analyses were performed to establish the relation between SJRP´s strains and previously described HRSV genotypes deposited in Genbank and further selection pressure analysis was performed to examine the replacement behavioral patterns of G protein ectodomain encoded amino acids.

## 2. Results and Discussion

### 2.1 HRSV Epidemiology and Cohorts Characterization

Brazil is a country of large territorial extension, but few studies have evaluated HRSV circulation patterns and genotypes, which are limited to hospital-based studies performed at Southeast and Northeast regions [20,21,22,24,25,26,22,24]. Studies including children that attended day care centers have been done mainly in Scandinavia, the United States and England [27], and evidenced that children who attended day care centers from the beginning of infancy present higher risks of respiratory infections comparing to children that did not attend day care centers [28]. Based on these works and due to the lack of informative data, we aimed to understand features, such as the diversity, genotypes and adaptation of HRSV strains circulating in two communities of São José do Rio Preto-SP, Brazil: children that attended a public day care center, and children that were hospitalized due to respiratory infections.

The day care center cohort was composed of 231 children, aged 1 to 78 months (average age of 30.85 months), 44.5% female and 55.5% male, which presented an HRSV frequency of 7.7%. From July 2003 to April 2004, this pathogen was mainly detected and isolated on winter and spring seasons, while in 2005, outbreaks were observed in late autumn and winter seasons. These results contrasts with the previously reported HRSV frequency of 29% [29], detected between May 2004 and September 2005 in clinical samples derived from the hospital, which was composed of 272 children including 57% male and 43% female, whose ages varied between 1 to 68 months. HRSV hospital outbreaks were observed in winter and spring 2004 and autumn 2005 [29].

Such variations on HRSV circulation patterns in both communities may have occurred due to environmental factors such as temperature or relative air humidity, co-circulation and competition with other respiratory pathogens as reported previously in São José do Rio Preto [29] or even due to virulence features as high infectivity and limited antigenic diversity of HRSV strains [30].

Episodes of respiratory infection, on HRSV positive children from the day care center, were characterized by the absence of severe symptoms and were generally limited to upper airways, whit no need of hospitalization during the respiratory infection. The most frequent symptom was runny nose (93.2%), followed by cough (58.2%), nasal obstruction (14.6%), wheezing (3.7%) and fever (2.4%). In contrast, HRSV positive children from the hospital, developed severe diseases such as pneumonia (24.1%), bronchiolitis (64%), and acute wheezing (16.2%) [29]. The most frequent symptoms were cough (93.1%), fever (91%) and coryza (62.1%) and nasal obstruction (50%) [29]. It is evident that strains isolated from the hospital were more pathogenic, accounting for severe symptoms, thus suggesting that multiple lineages co-circulated in São José do Rio Preto.

### 2.2 Phylogenetic Analysis

In order to understand the diversity and establish the relation between SJRP HRSV strains, we performed phylogenetic analyses of partial sequences obtained from the C-terminal ectodomain of the attachment protein (G) gene (n = 60). Results showed that SJRP´s HRSV strains were grouped into three distinct clusters (Supplementary Figure 1) and evidenced the co-circulation of multiple HRSV antigenic groups and genotypes, as reported in several regions of Brazil and São Paulo State [20,21,25,29].

**Figure 1:**
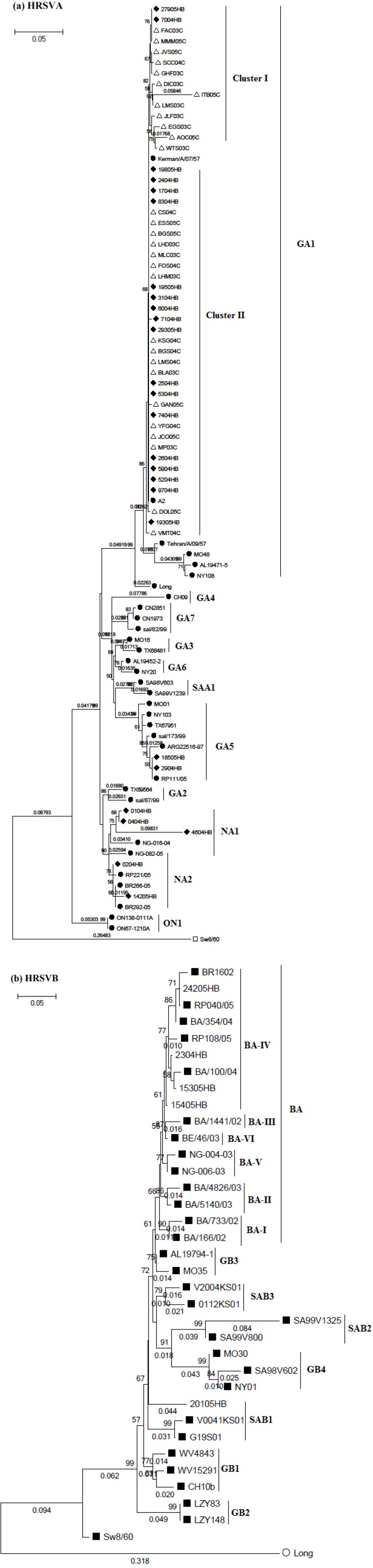
(**a**) Phylogenetic tree of José do Rio Preto-SP (SJRP) HRSVA nucleotide sequences of 265-270 in length, from the second variable region of the G gene. Reference strains representing known genotypes are indicated by a solid circle. SW8/60 (subtype B) was used as outgroup, marked by an open square (**b**) Phylogenetic tree of SJRP HRSVB nucleotide sequences from the second variable region of the G gene. Reference strains representing known genotypes are indicated by a solid square. Long (subtype A) was used as outgroup, marked by an open circle. Multiple sequences alignment and phylogenetic tree was constructed using Clustal W and Neighbor-joining method running within MEGA 5.05 software. Tree topology was supported by bootstrap analysis with 1000 pseudo replicate datasets. Bootstrap values greater than 50 are shown at the branch nodes. HB refers to strains isolated from the hospital (indicated by a solid lozenge) and C refers to strains isolated in the day care center (indicated by an open triangle).

Therefore, we proceeded to an analysis of SJRP´s HRSV sequences along with 32 HRSVA and 30 HRSVB reference strains derived from Genbank (Supplementary Table 1), including sequences derived from HRSV isolated in Brazil. The analysis confirmed the co-circulation of both HRSVA and HRSVB antigenic groups, however, most of the sequences (n=55) were clustered to HRSVA group, while five sequences were associated to HRSVB group (Figure 1). Oliveira and collaborators (2008) also reported a predominance of HRSVA strains over HRSVB in Uberlândia-MG, which agrees with most of the studies performed to identify on antigenic group subtypes, while Cintra and collaborators (2001) have found higher frequencies of both antigenic groups circulating in Ribeirão Preto-SP. Although Ribeirão Preto, Uberlândia and São José do Rio Preto are nearly located (between 200–300 km of distance), different geographical and demographical characteristics must be accounted and also, HRSV antigenic group analysis were performed in different seasons and years, which may contribute for differences on the HRSV subgroups detection.

It has been widely recognized that both subtypes circulate concurrently [31]. Zlateva and collaborators (2004) found the presence of multiple identical sequences among Belgian isolates, which suggested that certain strains predominated in a given epidemic season. Peret and colleagues (2000) also found a dominance of HRSVA over HRSVB, and predominance of 1–2 genotypes in five communities [10]. The predominance of HRSV-A viruses has been attributed to the higher variability among the HRSV-A strains [32]. Usually, the dominant strains shift yearly, suggesting a mechanism for frequent re-infections by evasion of immunity induced by previous strains [33].

Genotype classification through phylogenies demonstrated that SJRP HRSVA isolates, derived from both communities, were more related to GA1 (n=48), while GA5 (n=2), NA1 (n=3), NA2 (n=2) were detected only in samples from hospitalized children (Figure 1a). These results contrast with previous reports, which showed major prevalence of GA2 genotype during 1999 in Salvador-Ba [21], and during 2004 in Campinas-SP [24]. Interestingly, three Brazilian HRSV reference sequences (RP221/5, BR266-05 and BR292-05), circulating in São Paulo State during 2005 epidemic season, that have been previously described as GA2 genotypes, were clustered together with NA2, a recently identified genotype in Japan, that is genetically close to GA2 [12]. The fact that the three Brazilian strains may be more related to NA2 genotype is supported by the absence of NA2 sequences on phylogenetic analysis performed at the time they were reported, thus contributing to the association with GA2 genotype.

Indeed, inter-continental circulation of HRSV had been reported [14]. Viruses of the BA-I genotype circulated extensively in Buenos Aires from June to August of 1999. In December of the same year, the first BA-I sequence with an exact copy of the duplicated 60 nucleotide segment on the third hypervariable domain of G gene, from a non-Argentinean sample, was found in Belgium. Thus, it is clear that HRSV crossed the Atlantic (in either direction) in a period of few months [14], supporting that NA2 strains may have circulated in Brazil and Japan.

There was a strong phylogenetic association (bootstrapping value of 75%) between sequences obtained from GA1 isolates MMM05C and FAC03C from the day care center with the isolates 7004HB and 27905HB from the hospital. SJRP GA1 isolates were further divided into two major clusters, I and II, which suggested that individual lineages of GA1 genotype could be co-circulating in the period of analysis (Figure 1a), as also proposed by Eshaghi and collaborators (2012), that identified two NA1 lineages circulating in Ontario, Canada.

HRSVB strains were classified as SAB1 (n=1) (Figure 1b), also reported by Botosso and collaborators (2009) in São Paulo-SP and BA-IV (n=4). Although, BA genotype had been isolated in Brazil [23], this is the first time HRSVB strains are associated to BA-IV, which was also identified in Buenos Aires, Argentina in 2004 epidemic season, Quebec, Canada through 2001-2003, Kenya in 2003, Belgium in 1999, 2001-2003 [14] and most recently in China through 2006-2009 epidemic seasons [16].

### 2.3 Molecular Analysis of HRSV GA1 Genotype

**Figure 2:**
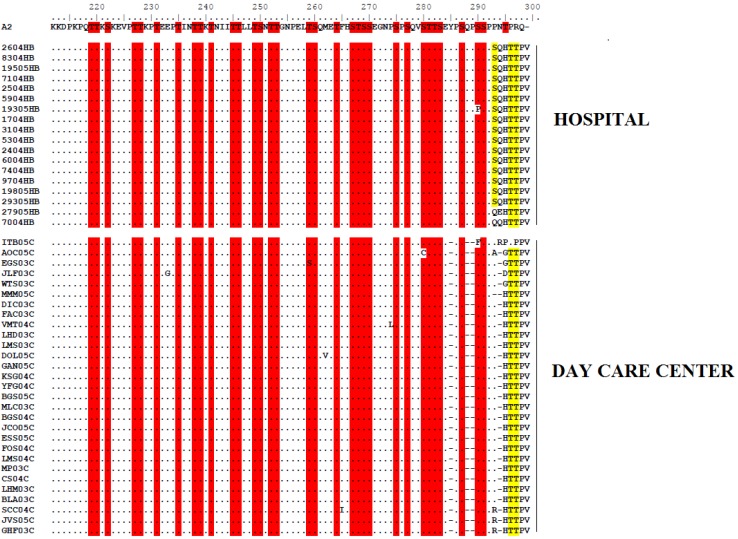
Alignment of deduced amino acid sequence of the G protein of HRSV GA1 genotype strains isolated in São José do Rio Preto-SP. Alignments are shown relative to the sequence of prototype strain A2. The amino acids shown correspond to positions 212 to 298 of the second hypervariable region of HRSVA strain A2 G protein. The alignment was done by the Clustal W algorithm running with BioEdit. Identical residues are represented as dots. Potential O-glycosylation sites conserved between HRSVA strain A2 and SJRP GA1 isolates are shaded in red. Potential O-glycosylation sites present only on SJRP GA1 isolates are shaded in yellow.

By comparing the nucleotide composition and the pattern of mutations among the 60 HRSV isolates, remarkable genetic flexibility could be observed, as previously noted worldwide [31,34,35,36]. However, two major clusters comprising several groups of identical sequences were identified only among the GA1 genotype strains, thus further analysis were carried out on this group due the lack of sampling of other genotypes. The alignments of nucleotides of the second hypervariable region of HRSV G gene from representative isolates for each GA1 group, compared to the HRSVA reference strain A2 (originally isolated in Australia in 1961), showed possible deletion/insertion sites on nucleotide positions 853, 854, 864, 865, 866 and 879 of the day care center strains, and on nucleotide position 877 of hospital strains (Supplementary Figure 2). Consequently, these variations accounted for changes on the deduced amino acid sequences of SJRP´s GA1 isolates, compared to A2 strain amino acid sequence. High variability of day care center sequences was observed mainly from residue 285 to 299, contrasting to hospital sequences which present high variability from residue 293 to the 299 (Figure 2). Such diversification is expected since a high level of genetic variation may be associated with the fact the G protein plays a key role in facilitating re-infections in HRSV—allowing evasion from cross-protective immune responses—and hence in the fluctuating patterns of viral circulation [25].

Since the two variable regions of the ectodomain contain high serine and threonine residues, which are potential acceptor sites for O-linked sugars affecting the antigenic structure of the G protein as well as impacting on virus infectivity [18,19], we performed an O-glycosylation site analysis on deduced aminoacid sequences from SJRP GA1 isolates. Third four sites were potentially O-glycosylated, among GA1 strains isolated from the day care center, while hospital GA1 strains retrieved third five potentially O-glycolsilated sites (G scores of 0.5–0.7) (Figure 2). Several amino acid positions that are likely to have O linked side chains (serine at 267, 270, 275, 283, 287 and threonine at 227, 231, 235, 253, and 282), reported by [37] were conserved in all SJRP GA1 isolates. SJRP GA1 strains lost the potential O-glycosilation site at amino acid position 295, nevertheless this resulted in an insertion of two potential O-glycosilation sites at positions 296 and 297. Furthermore, a potential O-glycosilation site insertion, at position 293, was only observed for hospital strains. It is possible that strains that have lost or changed O glycosylation can escape the immune system by losing recognition of a carbohydrate epitope [38]. We also performed N-glycosylation site analysis, which resulted in potential for N-glycosilation at sites 237 (G score of 0.6340) and 251 (G score of 0.5601). These sites were conserved among all SJRP sequences.

Eshaghi and colleagues (2012) found 21 and 27 potentially O-glycosylation sites in ON/RSV89 and ON/RSV181 (GA5 genotypes isolates) respectively, whereas an average of 33 sites were potentially O-glycosylated in NA1 isolates. By analyzing the same region, four putative N-glycosylation sites (Asn-X-Ser/Thr) were identified among Ontario circulating strains. Zlateva and collaborators (2004) predicted that serine residues at sites 117 and 290 are O glycosylated with a high potential and the positive selected threonine residue at site 225 was also predicted to be O- glycosylated (GA2 and GA5 genotypes).

Virus infectivity has been shown to be sensitive to the limited removal of N-linked or O-linked oligosaccharides [19]. Thus, sequence changes may influence the location of carbohydrate side chains, which are important determinants of the G glycoprotein antigenic structure [38]. Modifications observed on nucleotide sequences and consequently on the deduced amino acid residues composing the G protein ectodomain (Supplementary Figure 2 and Figure 2) of SJRP GA1 strains, also accounted for changes on potential O-glycosylation sites, thus suggesting that hospital and day care center strains could have different patterns of replication and virulence.

### 2.4 Selective Pressure

The second hypervariable region of HRSV G gene has been reported to be an important domain of diversity and adaptation between HRSV strains [17,25,38,39]. Therefore, in order to test neutrality deviations on SJRP GA1 strains and obtain first evidence of selection pressure action on this region of the G gene, we performed the Tajima's Neutrality Test [40], which resulted in D value: -2,32187 with a p-value < 0.001 and thus suggested the occurrence of sites under positive/purifying selection on GA1 genotype circulating in São José do Rio Preto (Table 1).

To test the hypothesis that the C-terminal hypervariable region of G proteins of SJRP´s GA1 isolates could be under action of selection pressure forces, we estimated the ratio of non-synonymous (dN) to synonymous (dS) substitutions per site across the multiple aligned dataset of all SJRP GA1 sequences, including all 32 HRSVA sequences downloaded from GenBank (Supplementary Table 1. We used the Datamonkey webserver [41] to calculate global and site-specific non-synonymous (d_N_) and synonymous (d_S_) nucleotide substitution rate ratios (ω = d_N_/d_S_) using the SLAC and MEME methods, which are based on a neighbor-joining generated phylogenetic tree and the best-fit nucleotide substitution model.

We observed a high average ratio of non-synonymous to synonymous nucleotide substitutions in the G glycoprotein gene (ω = 0. 0.645), however this value does not surpass the threshold of ω > 1, and it is therefore not indicative of positive selection [42]. Since the average ω is usually not sensitive enough to detect Darwinian selection at the molecular level [38], we used codon substitution models to detect sites under positive selection. Notably, amino acid residue 274 was the only site found to be subject of robust positive selection (p<0.05) by both methods, with posterior probability of 1.000 (100%). In fact, previous works [13,25,43] found several sites under adaptive evolution within the G protein, including amino acid residue 274, which is one of the sites that defines genotypes and lineages within genotypes, and correlate well with known epitopes described in escape-mutants selected with specific Mabs [44,45]. HRSV escape mutants that differ in their last 81 residues from the canonical Long prototype protein sequence, retain their compositions and hydropathy profiles, strongly suggesting that there may be indeed structural restrictions to changes in the G protein [25]. According to previous works [13,38,43], the HRSV evolution is driven by positive selection operating at specific codon positions and the fact that we identified only one site under positive selection could be due to the method we used to identify positive selected sites, since these studies carried out the analysis on the PAML program.

**Table 1:** Nucleotide diversity (π), Tajima D test (D), genetic distances (d) and statistical significance for Tajima D test (p) of identified human respiratory syncytial virus (HRSV) strains and GA1 genotype.

HRSV	N	Π	D	d	p
**A**	55	0.03465	-2.14299	0.048	p<0.05
**B**	5	0.03878	-0.85185	0.040	p>0.10
**GA1**	48	0.00924	-2.32187	0.010	P<0.01

## 3. Experimental Procedures

### 3.1 Cohorts and Clinical specimens

A total of 1,089 respiratory samples were obtained from children aged between 0 and 6 years in the period of July 2003 to September 2005. Two cohorts were included, one composed of children that were hospitalized at São José do Rio Preto Public Hospital (HB) (272 samples), presenting lower respiratory tract diseases (such as bronchiolitis and pneumonia), and another composed of children presenting acute respiratory infections (ARI) (characterized by single or combined occurrence of the following physical signs and symptoms: cough, pharyngitis, rhinorrhea, nasal congestion, headache, low grade fever, facial pressure and sneezing), that attended the Maria Ines Arnal Day Care Center (C) (817 samples) in São José do Rio Preto-SP, Brazil. Nasopharyngeal washes were obtained after instillation of 0.5ml of sterile PBS (Phosphate Buffered Saline - NaCl, Na2HPO4, NaH2PO4) into each nostril with immediate aspiration through a sterile neonatal canula inserted into the child’s nasopharynx. The sample was transferred to a sterile vial and immediately transported to the laboratory, processed and frozen at –80°C in Trizol LS (Invitrogen, Carlsbad, CA) for later RNA extraction and RT-PCR testing.

### 3.2 Viral detection and Nucleotide Sequencing

Total RNA was extracted from nasopharyngeal washes using guanidinium isothiocyanate phenol (Trizol LS, InvitrogenH, Carlsbad, CA) according to the manufacturer’s instructions. Reverse transcription was performed with High Capacity cDNA Archive Kit – Applied Biosystems (USA) according to the manufacturer’s instructions.

Partial HRSV G gene amplification was performed by a semi-nested PCR procedure. First, cDNA was amplified using the primer FV: 5´- GTTATGACACTGGTATACCAACC-3´ (based on sequences complementary to nucleotides 186 to 163 of the F protein gene [46] – and the forward primer GAB: 5`- YCAYTTTGAAGTGTTCAACTT-3´ (G gene, 504–524 nt). A semi-nested PCR was then performed with primers F1AB 5`- CAACTCCATTGTTATTTGCC-3´ (F gene, 3–22 nt) and GAB [9,10]. PCR assay was carried out in a reaction mixture containing 200ng of cDNA, 1 mM MgCl2, 0,2 mM dNTPs, 20 pmol of each primer, 2 U of Taq DNA Polimerase (Biotools, ESP) in a final volume of 50 μL. Amplification was performed in a GeneAmp PCR System 9700 thermocycler (Applied Biosystems Inc.) with the following parameters: 95ºC for 5 minutes, followed by 40 cycles of 1 min at 94ºC, 1 min at 55ºC and 1 min at 72ºC, and finally 7 min of extension at 72ºC.

The semi nested PCR was carried under the same conditions, with 20 pmol of each primer on a final volume of 50 μL. Both cDNA synthesis and PCR followed strict procedures to prevent contamination, including redundant negative controls and segregated environments for pre- and post-amplification procedures. Amplified products of the G gene were purified with a commercial kit (Qiagen PCR Purification Kit (Qiagen – USA)), according to the manufacturer’s instructions.

Sanger sequencing, of a 490-bp fragment of the G gene, was carried out with the same primer pair used for semi-nested PCR amplification on the ABI PRISM 3100 and 377 DNA sequencers (Applied Biosystems Inc.) using the BigDye Terminator v3.1 cycle sequencing kit (Applied Biosystems).

### 3.3 Phylogenetic Analysis

Sequences were assembled with the Sequence Navigator program version 1.0 (Applied Biosystems Inc., EUA) resulting in contigs of 265 nucleotides on average, to isolates from the day care center, and contigs of 270 and 330 nucleotides on average to isolates from the hospital. These contigs were compared to HRSV G gene nucleotides from reference strains representing different HRSVA and HRSVB genotypes identified in other cities or states of Brazil, South America, South Africa as well as HRSV sequences from other countries available at GenBank (Supplementary table 1). Multiple sequence alignments of the obtained fragments compared to globally sampled reference strains were performed using the Clustal W algorithm [47] and Bioedit sequence alignment-editing software. Phylogenetic associations were determined using Maximum Likelihood (ML) and Neighbor-Joining (NJ) methods running with MEGA 5.05 software [48]. Node support of each clade was evaluated using bootstrap analysis (1000 replicates) and the evolutionary distances were derived using the Kimura-2 parameter method [49].

### 3.4 O-glycosylation and N-glycosilation site analysis

Potential O-glycosylation and N-glycosylation sites were predicted using and NetOGlyc 3.1 [50] and NetNGlyc 1.0. The deduced AA sequences of the second hypervariable region of HRSV GA1 genotypes (encompassing AA 212 to the end of the G protein) were compared to those of HRSV-A2 strain.

### 3.5 Selection pressure analysis

Neutrality Tajima´s D test [40] was performed by DnaSP version 5.10.01 software [51] to verify statistically significant deviations among sequences and provide first evidence of selection acting on SJRP HRSV isolates. 

To determine the selection pressures acting on the ectodomain of the G gene of GA1 genotype isolates, we estimated the numbers of non-synonymous (dN) to synonymous (dS) nucleotide changes per site; when dN>dS, this was indicative of positive selection. Site-specific (that is, codon-specific) selection pressures were determined using the Single Likelihood Ancestral Counting (SLAC) available in the HyPhy package [52] and Mixed Effects Model of Evolution (MEME) [53] methods, accessed through the Datamonkey webserver [41]. These methods were run using best fit nucleotide model on a neighbor-joining phylogenetic tree. Using this procedure, only codon 274 contained statistically significant evidence for positive selection.

### 3.6 Ethics Statement

A Written Consent signed by the parents or legal responsible guardians was obtained for each child. This study was approved by Research Ethics Committee from Unesp/IBILCE, Project Number 3777/2001 by opinion n° 062/2001 on June 11, 2001 in São José do Rio Preto, Brazil.

## Supplementary Files

Supplementary File 1: PDF-Document (PDF, 340 KB)
