# Supplementary material for: Diversity and Adaptation of Human Respiratory Syncytial Virus Genotypes Circulating in Two Distinct Communities: Public Hospital and Day Care Center
Source: Viruses. 2012 Oct 24;4(11):2432–47. doi: 10.3390/v4112432 (PMC3509657; doi:10.3390/v4112432)

Diversity and Adaptation of Human Respiratory Syncytial Virus Genotypes Circulating in Two Distinct Communities: Public Hospital and Day Care Center

Luiz Gustavo Araujo Gardinassi[3]*, Paulo Vitor Marques Simas[1*], Deriane Elias Gomes[2], Felipe Cavassan Nogueira[1], Caroline Measso do Bonfim[1], Gustavo Rocha Garcia[3] Claudia Márcia Aparecida Carareto[1], Paula Rahal[1], Fátima Pereira de Souza[2]**.

Supplementary Table 1 – HRSVA and HRSB Reference Strains used on multiple alignements and phylogenetic analysis derived from GenBank.

Antigenic Group (A or B)	Strain	Accession Number	Genotype
GROUP A	Long	M17212	Prototype
	A2	M11486	Prototype
	AL19471-5	AF233902	GA1
	MO48	AF233914	GA1
	NY108	AF233917	GA1
	Kerman/A/07/57	GU339399	GA1
	Tehran/A/09/57	HM063452	GA1
	TX69564	AF233923	GA2
	SAL/87/99	AY472086	GA2
	MO16	AF233913	GA3
	TX68481	AF233920	GA3
	CH09	AF065254	GA4
	MO01	AF233909	GA5
	NY103	AF233916	GA5
	TX67951	AF233919	GA5
	ARG22516-97	AY910841	GA5
	SAL/173/99	AY472094	GA5
	RP111-05	EU635820	GA5
	AL19452-2	AF233901	GA6
	NY20	AF233918	GA6
	CN2851	AF233907	GA7
	CN1973	AF233904	GA7
	SAL/82/99	AY472093	GA7
	NG01604	AB470478	NA1
	NG08205	AB470479	NA2
	RP221-05	EU635828	NA2
	BR266-05	EU582161	NA2
	BR292-05	EU582162	NA2
	SA98V603	AF348807	SAA1
	SA99V1239	AF348808	SAA1
	ON138-0111A	JN257694	ON1
	ON67-1210A	JN257693	ON1
GROUP B	Sw8/60	M55633	Prototype
	WV15291	M73542	GB1
	CH10b	AF065250	GB1
	WV4843	M73540	GB1
	LZY83	GU357513	GB2
	LZY148	GU357522	GB2

	AL19794-1	AF233925	GB3
	MO35	AF233929	GB3
	SA98V602	AF348824	GB4
	MO30	AF233928	GB4
	NY01	AF233931	GB4
	V0041KS01	AY226535	SAB1
	G19S01	AY226522	SAB1
	SA99V1325	AF348822	SAB2
	SA99V800	AF348821	SAB2
	V2004KS01	AY226536	SAB3
	0112KS01	AY226526	SAB3
	BA/733/02	DQ227374	BA-I
	BA/166/02	DQ227368	BA-I
	BA/4826/03	DQ227389	BA-II
	BA/5140/03	DQ227393	BA-II
	BA/1441/02	DQ227381	BA-III
	BA/354/04	DQ227406	BA-IV
	BA/100/04	DQ227395	BA-IV
	RP040/05	EU635852	BA-IV
	RP108/05	EU635861	BA-IV
	BR1602	HM021225	BA-IV
	NG-004-03	AB175819	BA-V
	NG-006-03	AB175820	BA-V
	BE/46/03	AY751110	BA-VI

Supplementary Figure 1. a) Phylogenetic tree of SJRP HRSV nucleotide sequences from the second variable region of the G gene.**b)** Phylogenetic tree of SJRP HRSV deduced amino acid sequences from the second variable region of the G gene Multiple sequences alignment and phylogenetic tree was constructed using Clustal W and Maximum Likelihood method running within MEGA 5.05 software. Tree topology was supported by bootstrap analysis with 1000 pseudo replicate datasets. Bootstrap values greater than 50 are shown at the branch nodes.

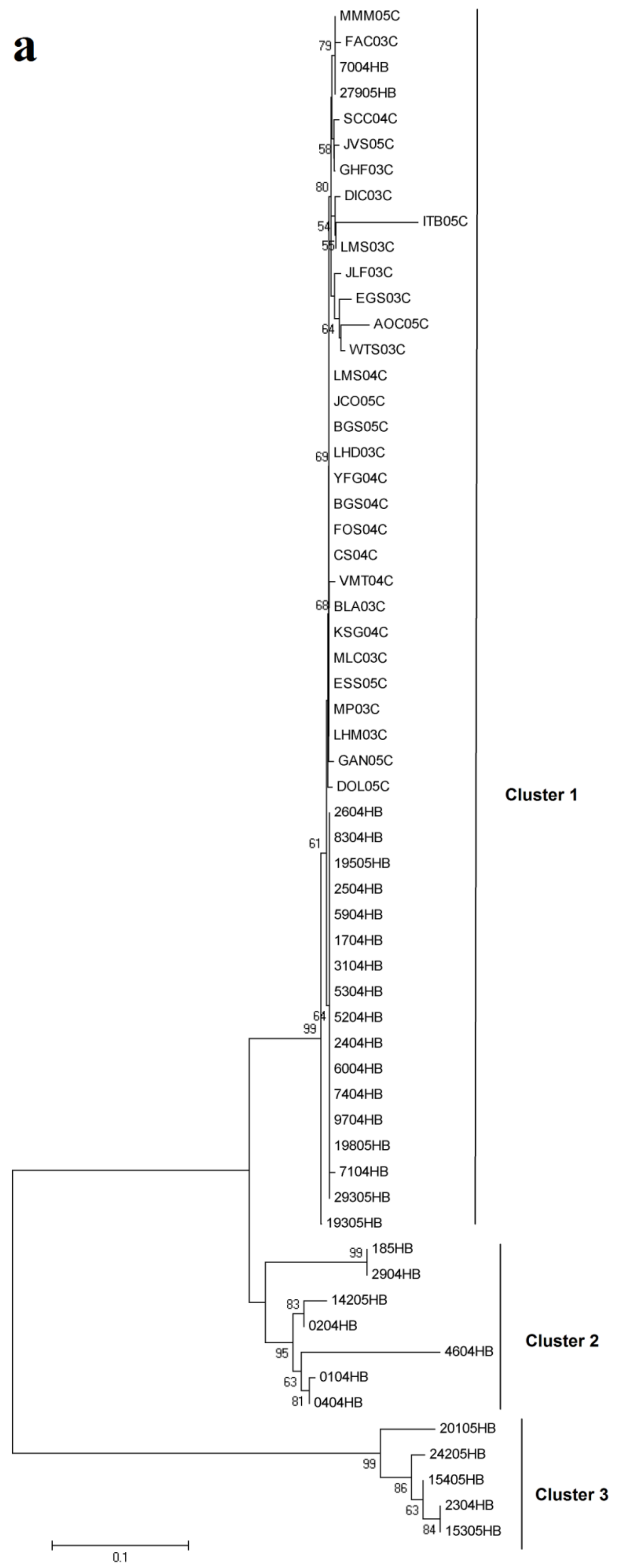
a
MMM05C
79
FAC03C
7004HB
27905HB
SCC04C
JVS05C
58
GHF03C
80
DIC03C
ITB05C
54
55
LMS03C
JLF03C
EGS03C
AOC05C
64
WTS03C
LMS04C
JCO05C
BGS05C
69
LHD03C
YFG04C
BGS04C
FOS04C
CS04C
VMT04C
68
BLA03C
KSG04C
MLC03C
ESS05C
MP03C
LHM03C
GAN05C
DOL05C
2604HB
61
8304HB
19505HB
2504HB
5904HB
1704HB
3104HB
5304HB
64
5204HB
99
2404HB
6004HB
7404HB
9704HB
19805HB
7104HB
29305HB
19305HB
Cluster 1
99
185HB
2904HB
83
14205HB
0204HB
95
4604HB
63
0104HB
81
0404HB
Cluster 2
20105HB
99
24205HB
86
15405HB
63
2304HB
84
15305HB
Cluster 3
0.1

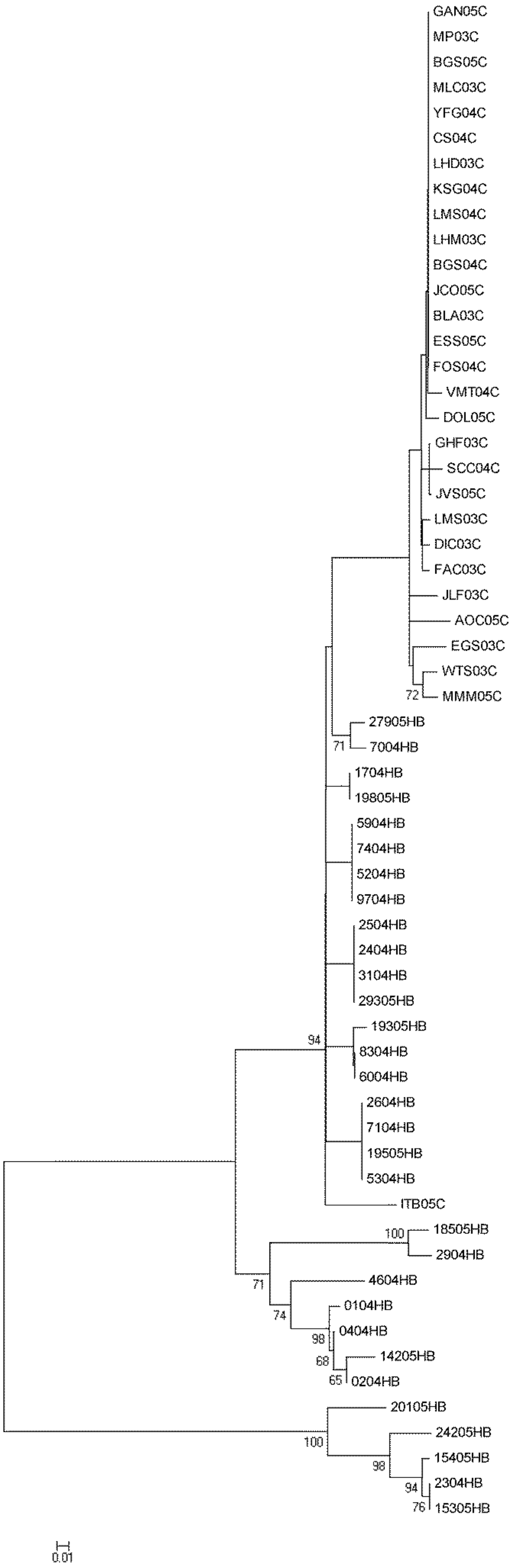
GAN05C
MP03C
BGS05C
MLC03C
YFG04C
CS04C
LHD03C
KSG04C
LMS04C
LHM03C
BGS04C
JCO05C
BLA03C
ESS05C
FOS04C
VMT04C
DOL05C
GHF03C
SCC04C
JVS05C
LMS03C
DIC03C
FAC03C
JLF03C
AOC05C
EGS03C
WTS03C
72
MMM05C
27905HB
71
7004HB
1704HB
19805HB
5904HB
7404HB
5204HB
9704HB
2504HB
2404HB
3104HB
29305HB
19305HB
94
8304HB
6004HB
2604HB
7104HB
19505HB
5304HB
ITB05C
100
18505HB
2904HB
4604HB
71
74
0104HB
98
0404HB
68
14205HB
65
0204HB
20105HB
100
24205HB
98
15405HB
94
2304HB
76
15305HB
0.01

Supplementary Figure 2. Alignment of partial nucleotide sequence of the G protein of HRSV GA1 genotype strains isolated in São José do Rio Preto-SP. Alignments are shown relative to the sequence of prototype strain A2. The nucleotides correspond to positions 801 to 898 of the second hypervariable region of HRSVA strain A2 G protein. The alignment was done by the Clustal W algorithm running with BioEdit. Identical nucleotides are represented as dots. Sites of Insertion/Deletion are shaded in gray.

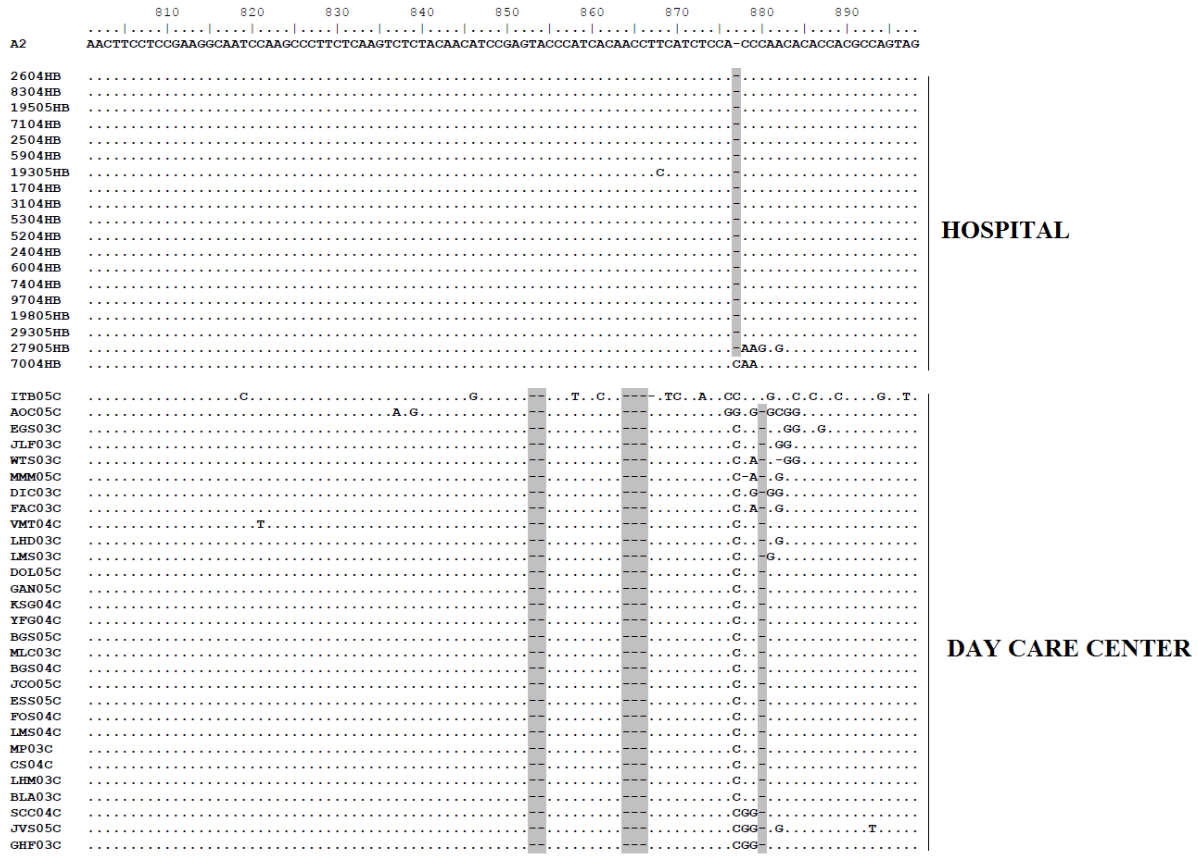

Supplement: Supplementary File 1: — PDF-Document (PDF, 340 KB) [file viruses-04-02432-s001.pdf]
